# Patient Profile and Management of Delirium in Older Adults Hospitalized Due to COVID-19

**DOI:** 10.3390/healthcare10040724

**Published:** 2022-04-13

**Authors:** Pablo Jorge-Samitier, Raúl Juárez-Vela, Iván Santolalla-Arnedo, Isabel Antón-Solanas, Vicente Gea-Caballero, Juan Luis Sánchez-González, María Teresa Fernández-Rodrigo

**Affiliations:** 1 Department of Physiatry and Nursing, Hospital Clínico Lozano Blesa, University of Zaragoza, 50009 Zaragoza, Spain; pjorge@salud.aragon.es; 2 Research Group GRUPAC C, Department of Nursing, University of La Rioja, 26004 Logroño, Spain; 3 Department of Physiatry and Nursing, University of Zaragoza, 50009 Zaragoza, Spain; ianton@unizar.es (I.A.-S.); maitefer@unizar.es (M.T.F.-R.); 4 Research Group Nursing Research in Primary Care in Aragón (GENIAPA) (GIIS094), Institute of Research of Aragón, 50009 Zaragoza, Spain; 5 Faculty of Health Sciences, Valencian International University, 46002 Valencia, Spain; vagea@universidadviu.com; 6 Community Health and Care Research Group, Faculty of Health Sciences, Valencian International University, 46002 Valencia, Spain; 7 Department of Nursing and Physiotherapy, University of Salamanca, 37008 Salamanca, Spain; juanluissanchez@usal.es; 8 Research Group: Water and Environmental Health, Research Institute in Environmental Sciences of the University of Zaragoza (IUCA), University of Zaragoza, 50009 Zaragoza, Spain

**Keywords:** COVID-19, coronavirus, SARS-CoV-2, acute delirium in older persons, acute confusional state, hospital

## Abstract

SARS-CoV-2 can cause neurologic symptoms, as well as respiratory ones. Older adults are at risk of developing acute delirium in older persons (ADOP). The combination of experiencing respiratory isolation due to COVID-19, as well as other associated risk factors for older adults, may have had an impact on ADOP and ADOP management in the acute hospital setting. This study aimed to analyze the characteristics of ADOP in patients admitted to a COVID-19 unit. An observational prospective study on a sample of 108 patients was carried out between November 2020 and May 2021. The following data were collected: sociodemographic characteristics, risk factors for ADOP, management of ADOP, and impact on ADOP on both functional and cognitive deterioration. A 29.6% proportion of older adults admitted to an acute COVID-19 unit presented hyperactive ADOP, mainly during the night. Management of ADOP in our sample involved mainly pharmacological treatment and had a serious impact on hospital stay and both functional and cognitive deterioration. Preventive strategies and being accompanied by a relative or a carer may be useful to manage ADOP during hospital admission due to COVID-19.

## 1. Introduction

SARS-CoV-2, a coronavirus responsible for a contagious disease causing severe acute respiratory syndrome known as COVID-19, was first diagnosed in December 2019. Initially limited to Wuhan (China), COVID-19 soon extended to other countries, including Spain and Italy [1,2]. The first COVID-19 cases in Spain were first diagnosed as early as February 2020. However, the disease soon extended to the whole country, causing a high rate of mortality and morbidity, especially among the elderly population. In this period, most patients admitted to acute COVID-19 units were aged 70 or older (70%), had multiple morbidities, and presented a variable degree of cognitive impairment and dependency. This had a serious impact on the healthcare service, which was soon unable to cope with the high demand for acute and intensive care [3,4].

SARS-CoV-2 causes an inflammatory lung reaction that frequently evolves to pneumonia, which is the primary cause of hospital admission. However, COVID-19 also causes other symptoms, including neurologic ones such as dizziness, nausea, vomiting, hypotension, asthenia, myalgia, headache, anosmia, ageusia, confusion, delirium, and, less frequently, ataxia, encephalitis, stroke, and convulsions. These sequelae can be evidenced histologically in the neural tissue of the COVID-19 patient [5].

In the elderly, the presence and combination of specific factors can cause acute delirium in older persons (ADOP). ADOP is characterized by temporospatial disorientation, psychomotor agitation, hallucinations, paranoia, as well as difficulty to stay alert, with a frequently sudden and fluctuating onset. ADOP can be classified as hyperactive, hypoactive, and mixed [6]. A range of factors are associated with an individual’s predisposition to experiencing ADOP. In addition, there are several precipitating factors which can trigger or contribute to ADOP [6]. These factors coincide with some of the most prevalent symptoms of COVID-19, including fever, cough, diarrhea, fatigue, and neurologic symptoms [2,5]. In fact, ADOP itself has been described as one of the initial symptoms of COVID-19 in patients with dementia [2]. The incidence of ADOP increases with age, reaching 20–30% in hospitalized patients aged 65 [7,8,9,10,11,12]. Usually, ADOP is associated with longer hospital admission, cognitive impairment, functional deterioration, and a higher rate of mortality [10,12,13,14], thus increasing healthcare expenditure and cost [11,15]. Several authors have investigated preventative and diagnostic ADOP models [16,17,18]. From the early days of the pandemic, it was observed that numerous older adults admitted to hospital due to COVID-19 presented predisposing and precipitating factors of ADOP [17,19]. These patients, already at risk of developing ADOP, were admitted to COVID-19 units where they could not be accompanied by a friend of relative, and healthcare staff were obliged to wear personal protective equipment (PPE), which hindered communication and interaction between patients and healthcare staff, caused fear, and contributed to the worsening of specific symptoms, such as disorientation and confusion [19,20]. This accentuated the feeling of social isolation, possibly contributing to increased use of physical restrictions, ADOP exacerbation and prolongation, increased pharmacological management of ADOP, and worse patient outcomes, including mortality [17].

According to previous investigations [1,2,12,21,22,23], 11–42% of older adults admitted to hospital due to COVID-19 developed ADOP, suggesting that an association may exist between COVID-19 and ADOP. The aim of this study was to analyze the incidence of ADOP in a COVID-19 special unit in a large tertiary hospital in the city of Zaragoza (Spain), as well as ADOP predisposing and precipitating factors, and ADOP progression and management.

## 2. Materials and Methods

A descriptive prospective study was carried out between 1 November 2020 and 29 May 2021. The end of the process of data collection coincided with the closure of the COVID-19 special unit in this hospital.

A convenience, consecutive sampling method was used to identify potential participants. We recruited every patient who gave their informed consent to take part in this investigation and met the selection criteria. Inclusion criteria to participate included: (1) patients aged 65 or over, admitted to a COVID-19 unit in a large tertiary hospital in Zaragoza (Spain), (2) being admitted for 3 days at least, and (3) being able and willing to sign the consent form during the first or second day of admission. We excluded patients who were admitted due to suspected COVID-19 but whose diagnosis could not be confirmed through a nasopharyngeal PCR test.

Data were collected using an ad hoc questionnaire comprising several validated tools classified into three sections. The first section comprised sociodemographic variables (age, gender, place of residence, and cohabitants) and predisposing factors of ADOP as described by different predictive models of ADOP [13]. In order to assess these factors, we analyzed comorbidities using the classification of the Andalusian Healthcare Service [24]. This scale is frequently used in Spain to assess comorbidity, defined as two or more chronic, potentially disabling conditions, namely, heart failure, renal failure, chronic hepatopathy, COPD, autoimmune disease, inflammatory bowel disease, chronic anemia, stroke, chronic neurologic disease, active neoplasia, osteoarticular disease, and advanced diabetes mellitus. In addition, we used the Barthel Index for Activities of Daily Living (ADL) in its Spanish version to assess patients’ functional deterioration [25]. The Barthel Index is used worldwide to assess ADL and presents high intra-observer reliability (kappa coefficient between 0.47 and 1.0) and high inter-observer reliability (kappa coefficient between 0.84 and 0.9). The Barthel Index evaluates 10 variables, including presence or absence of fecal or urinary incontinence, and help needed with grooming, toilet use, feeding, transfers (e.g., from chair to bed), walking, dressing, climbing stairs, and bathing. The total score ranges from 0 to 100, with a value of 0 indicating total dependency for the ADL, and a score of 100 indicates total independency. Generally, the level of dependency is divided into five categories (independent, slight dependent, moderate dependent, severe dependent, total dependent). However, for the purpose of the statistical analysis, we made the decision of grouping the categories total dependent and severe dependent (0–35), and the categories slight dependent and independent (>70), and maintaining the category moderate dependent (40–65). We also used the Pfeiffer Short Portable Mental Status Questionnaire (SPMSQ) in its Spanish version [26] to assess cognitive impairment. The SPMSQ comprises ten questions dealing with orientation, personal history, remote memory, and calculations. Total score ranges from 0 to 10. Results are classified into three categories, namely, normal (0–3), slight–moderate cognitive impairment (4–7), and severe cognitive impairment (8–10). Inter- and intra-observer reliability is 0.738 and 0.925, respectively. Sensory deficit (visual and auditive) and previous history of ADOP were assessed through the interview and the patient’s clinical history. Nutritional and hydration status were assessed using the last section of the Norton scale modified by the INSALUD [27], assessing general physical state. The whole scale has a sensitivity of 84% and a specificity of 83%. Specifically, the general physical scale section measures body mass index (BMI), daily calorie intake, and skinfolds, and classifies nutritional status into good, average, mediocre, and bad. Finally, a patient was considered to have a sleep disorder if they consumed hypnotics daily.

Data comprised in the second section were collected only if patients experienced ADOP during hospital admission. ADOP was confirmed though the Confusion Assessment Method (CAM). Sharon K Inouye et al. published in 1990 “Clarifying Confusion: The Confusion Assessment Method”, where she presented the CAM, a tool to diagnose delirium. Later, in 2008, Inouye et al. published “The Confusion Assessment Method: A Systematic Review of Current Usage”, demonstrating a sensibility of 94% (confidence interval 91–97%) and specificity of 89% (confidence interval 85–94%). Since then, many studies have used this information to justify using the CAM as tool to diagnose delirium [28,29] worldwide [30]. The CAM diagnostic algorithm comprises four features. Feature 1 assesses acute onset or fluctuating course of delirium through the identification of evidence of an acute change in mental health status from the patient’s baseline, whilst feature 2 measures inattention, defined as difficulty focusing attention, being easily distractible, or having difficulty keeping track of what is being said. If both features are confirmed, then features 3 and 4 are subsequently evaluated. Feature 3 assesses disorganized or incoherent thinking, characterized by rambling or irrelevant conversation, unclear or illogical flow of ideas, or unpredictable switching from subject to subject, whilst feature 4 assesses the patient’s level of consciousness, which is classified into alert (normal), vigilant (hyperalert), lethargic (drowsy, easily aroused), stupor (difficult to arouse), or coma (unarousable). ADOP is confirmed if both features 1 and 2, and either feature 3 or feature 4, are identified or positive [30]. Total score for the CAM test ranges from 0 to 4, with scores of 3 or 4 suggesting a diagnosis of ADOP. The CAM tool can be easily and quickly administered by adequately trained nursing staff [31]. After completing the CAM assessment, potential precipitating factors of ADOP were collected, including those associated with hospital admission, such as hospital stay, routine and sleep changes, and increased visual and auditive stimuli. In addition, we recorded interventions to manage ADOP, including verbal and behavioral contention, frequently implemented by nursing staff, pharmacological interventions, led by medical staff, and physical contention, in order to reduce the risk of potential complications [28,32].

Finally, the third section was completed at the time of patient discharge and comprised data regarding the patient’s clinical evolution during hospital admission. Specifically, data regarding precipitant factors of ADOP were collected, namely, intravenous (IV) cannula, IV therapy, oxygen therapy, urinary catheter, raised bed rails, diapers, pain and/or discomfort, fever, and metabolic disorders. In addition, the Barthel Index and the SPMSQ were completed again at this point. Finally, information was collected regarding discharge destination, namely, home, healthcare service, and hospital exitus.

Information was collected by experienced nursing staff who were familiar with the processes of data collection, except for the CAM questionnaire, for whose completion they were appropriately trained.

Statistical analyses were completed using SPSS-25 (IBM, Armonk, NY, USA). A *p*-value less than 0.05 was considered to be statistically significant for all the analyses. Descriptive statistics, including frequency (mean and standard deviation) and percentages, were used to describe sociodemographic and clinical variables. The association between the study variables and ADOP was analyzed using the Chi-square test, except when the number of cases was fewer than 5, in which case Fisher’s exact test was applied.

All the participants, and their next of kin if the patients were cognitively impaired, were informed about the study aims and requirements. Confidentiality was guaranteed according to the Organic Law 3/2018, of 5 December, of Data Protection.

## 3. Results

A total of 108 older adults took part in this investigation. Overall, 61.1% were over 75, and 20.4% were older than 85. Almost half of the participants were women, and around half lived in rural areas. Most of the participants lived at home with their partner, a relative, or a paid carer; only 13.9% of older adults lived in a nursing home. With regard to the participants’ clinical history, 52.8% had comorbidities, including cardiovascular and respiratory chronic pathologies. In addition, 13.8% had dementia, including Alzheimer’s, vascular dementia, or stroke, resulting in a variable degree of dependency (Table 1).

A total of 32 participants (29.6%) had ADOP during hospital admission due to COVID-19. A total of 28 (87.5%) older adults with ADOP experienced hyperactive dementia, 3 experienced hypoactive dementia, and 1 presented mixed dementia. An 81.2% proportion of the patients with ADOP were over 75 and they were mostly women, while 65% had comorbidities, 41.7% were moderately or severely disabled, and 56.3% experienced moderate-to-severe cognitive deterioration. The percentage of patients with ADOP who were moderately or severely disabled and who presented moderate or severe cognitive impairment was much higher than in the group of older adults who did not develop ADOP during hospital admission (Table 2).

**Figure 1:**
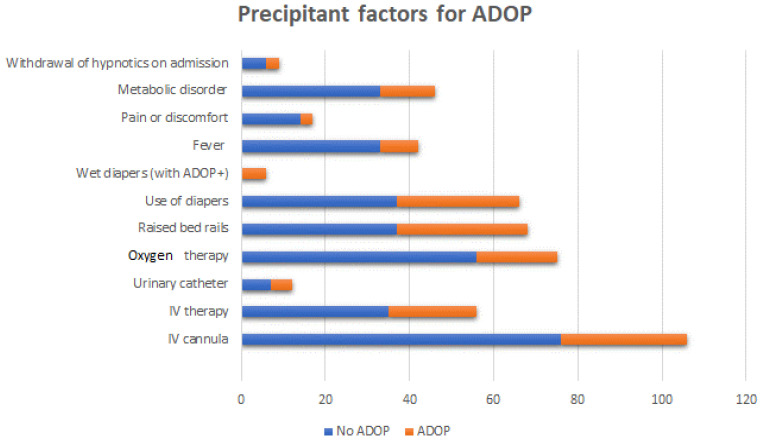
Precipitant factors for ADOP.

Overall, 68.8% of patients with ADOP presented symptoms, mainly at night. Among cases of ADOP, 84.4% were diagnosed during the first week, 43.8% during the first day, and 15.6% during the second day of admission. Precipitant factors of ADOP in our sample are presented in Table 3 and Figure 1.

Verbal and behavioral contention was needed only in seven patients with ADOP (22%). A total of 19 patients with ADOP (60%) required pharmacological treatment, of whom 3 (9%) received physical contention. No intervention was implemented in case of hypoactive ADOP. The most frequently used drugs to manage ADOP were haloperidol (50%) and quetiapine (32%), and, more rarely, lorazepam, olanzapine, and risperidone. Table 4 studies the possible association between management of ADOP and functional and cognitive deterioration, age, and gender.

**Figure 2:**
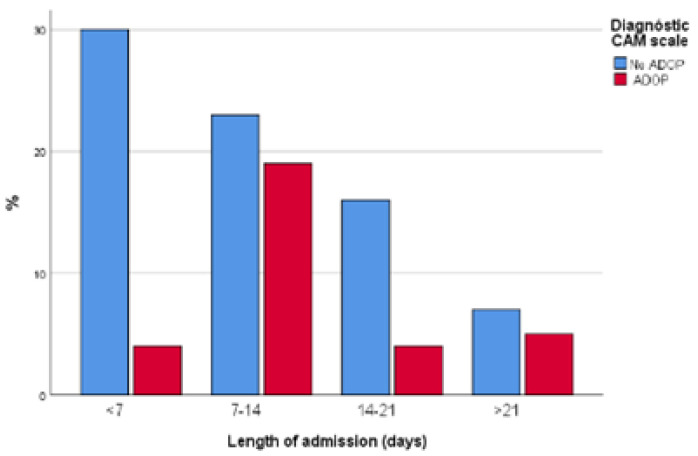
Length of admission.

**Figure 3:**
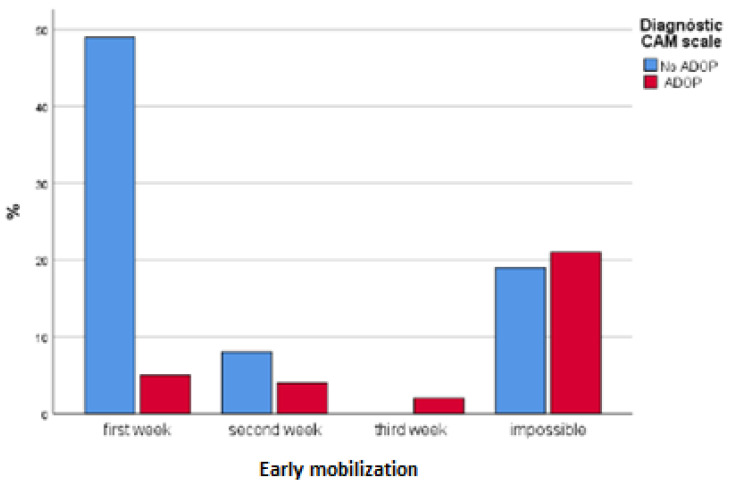
Early mobilization.

**Figure 4:**
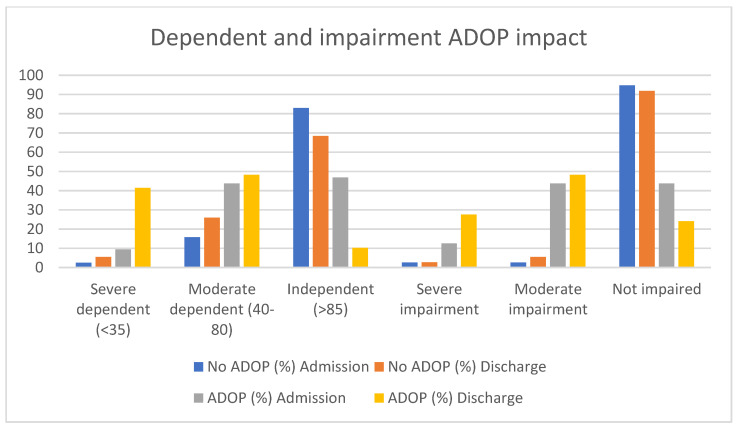
Dependent and impairment ADOP impact.

Sometimes, ADOP management was not effective, or symptoms of ADOP were recurrent, which impacted on the duration of pharmacological treatment for ADOP. A total of 31.6% of patients with ADOP received pharmacological treatment for 1 day, whilst 63.2% received pharmacological treatment for at least 3 days. Three patients with ADOP had to be restricted physically. The duration of physical containment in these cases ranged from 1 to 10 days. There were no statistically significant differences between groups in terms of discharge destination. Length of hospital stay, early mobilization, and the results from the Barthel Index and Pfeiffer’s SPMSQ are presented in Table 5 and Figure 2, Figure 3 and Figure 4.

## 4. Discussion

Overall, 29.6% of the older adults admitted to hospital for the primary reason of COVID-19 developed ADOP. Our results are similar to those reported by previous investigations carried out throughout the year 2020. A systematic review by Aguilar et al. [29] comprising 43 articles concluded that prevalence of ADOP in COVID-19 patients ranged from 20 to 30%. Garcez et al. [21] and Mendes et al. [22] reported similar prevalence rates, 30% and 20.4%, respectively, whilst another study by Ticinesi et al. [1] identified a much lower percentage of ADOP amongst patients admitted with COVID-19.

A total of 5.5% of our participants died during hospital admission. Although the mortality rate during admission was higher in the group of patients with ADOP (9.9%), no significant differences were found between both groups. Our findings differ from those reported by Garcez et al. [21], Ticinesi et al. [1], and Mendes et al. [22]. This may be due to the fact that we did not collect data on mortality after patients had been discharged to a critical care unit. As in a previous study [2], 25.7% of our participants were discharged to a critical care unit.

Symptoms of ADOP manifested mainly at night (68.8%) [7,8,9,10,11,12,13,14,15]. The vast majority of patients with ADOP presented hyperactive delirium. Our rates of hypoactive and mixed delirium are slightly lower than those reported in previous studies by Mendes et al. [22], McLoughlin et al. [12], and Poloni et al. [2], who reported higher rates of hypoactive delirium (37–52.4%), lower rates of hyperactive delirium (35–53%), and slightly higher rates of mixed delirium (23%). It is possible that our lower rates of hypoactive and mixed delirium are due to the inability of the healthcare staff to identify these types of delirium, as has previously been reported in the literature [14,33,34].

We identified several predisposing factors of ADOP, namely, age, comorbidity, and cognitive and functional deterioration. It is important assess these factors when assessing the risk of ADOP in older adults admitted to hospital [6,17,18,25]. Generally, being male is a predisposing factor of ADOP [9,35,36]. However, we did not find significant differences between men and women in the number of cases of ADOP. Similarly, no statistical differences were identified between nutritional status and hypnotic use, and ADOP, during hospital admission in our sample. Similar to previous studies [36,37], cognitive and functional impairment was inversely associated with ADOP in hospitalized older adults with COVID-19.

Regarding ADOP management, 25% of our patients with ADOP were managed using verbal and behavioral contention measures, whilst a larger percentage of cases required pharmacological treatment, with only a small minority requiring measures of physical restraint. Verbal and behavioral contention measures were most effective when patients were not functionally and cognitively impaired, were women, and younger than 85. Pharmacological treatment for ADOP was administered mainly to patients with a slight-to-moderate level of functional and cognitive impairment, not finding any gender or age differences in our sample. It is worth highlighting that lorazepam was prescribed twice for the management of ADOP in our sample, despite it not being recommended by recent investigations [7,28,38], expect in the case of alcohol or benzodiazepine withdrawal. It is likely that, in these cases, lorazepam was withdrawn on admission and prescribed again when symptoms of ADOP manifested at a later stage. In our study, pharmacological contention measures were usually maintained for at least three days, whilst physical restraints were maintained for up to 10 days in one case. According to O’Hanlon et al. [17] and Nikooie et al. [29], antipsychotic management of ADOP is not always effective, and it is not without risks [20]. Yet, as demonstrated in our study, pharmacological management of ADOP is still frequent [38].

There was a significant association between ADOP and length of admission. Our results coincide with those reported by Aguilar et al. [29], who demonstrated association between ADOP prolonged hospital stay and increased mortality. Mendes et al. [22] did not find an association between length of admission and ADOP, but they did observe an association between mortality and ADOP. Our findings indicate most older adults with COVID-19 suffered a variable degree of both functional and cognitive deterioration during hospital admission. However, functional and cognitive impairment was higher in the cohort of older adults with COVID-19 who developed ADOP. Similar findings were observed by Garcez et al. [21] and Aguilar et al. [29], who established an association between severe COVID-19 and neurologic symptoms, and Mao et al. [5], who observed that 20–30% of patients admitted to hospital with COVID-19 experienced ADOP and 36% developed neurologic symptoms. According to Mendes et al. [22] patients who had cognitive deterioration were four times more likely to develop ADOP; the risk of ADOP during hospital admission also increased in case of functional impairment. However, findings are inconsistent [12].

ADOP symptoms manifested mainly during the night, soon after admission [16,18,33,37,39]. It is therefore important to implement preventative measures at an early stage. According to previous investigations [29], as much as 30–40% of cases of ADOP could be prevented. However, assessment of the risk of ADOP, and therefore also implementation of preventative measures, is not common practice in many wards and hospital units [17,20]. Early mobilization has been described as an effective measure to prevent functional and cognitive deterioration and ADOP in hospitalized older adults [29]. Our findings support this argument, as patients who mobilized early were less likely to experience ADOP. However, early mobilization was not always easy to achieve during the pandemic as patients were, for the most part, unaccompanied due to COVID-19, and time of direct patient contact was also limited for the same reason.

Our study has some weaknesses that we wish to acknowledge. Firstly, data collection took place in November 2020, during a period which was later described as the “third wave” in the region of Aragon. By this period, nursing homes were more efficient at implementing social distancing and isolation measures, which contributed to decreasing the number of institutionalized older adults who were admitted to hospital for the primary reason of COVID-19. In addition, vaccination campaigns started in January 2021, contributing to reducing the number of infections, the severity of the symptoms, and thus the number of hospital admissions, in older adults. This had an impact on the age profile of patients admitted to hospital due to COVID-19, who were younger and had fewer predisposing factors for ADOP, than those admitted earlier in 2020. Finally, our sample size was relatively small, which limited our ability to establish significant differences between ADOP and non-ADOP patients, even though our findings do coincide with those reported in previous investigations.

## 5. Conclusions

The prevalence of ADOP in older adults admitted to hospital for the primary reason of COVID-19 in our study is high, and it is similar to that reported in previous investigations. Pharmacologic measures to manage ADOP were frequently implemented in our sample; physical restraint was also used in some cases. Once initiated, these measures were maintained for a variable length of time in most cases. This may have contributed to worsening functional and cognitive deterioration during hospital stay. A holistic approach to ADOP management is necessary to identify patients at risk and prevent ADOP. This approach may include measures such as ADOP screening, early mobilization, non-pharmacological interventions, and specific training of both formal and informal carers and healthcare professionals and companies. Although there may be barriers for the implementation of these measures, we argue that they may be effective to improve patient outcomes in the medium-to-long term. Thus, adopting a patient (and his or her relatives)-centered care approach is essential in the management of ADOP.

## Figures and Tables

**Table 1:** Sociodemographic characteristics.

Sociodemographic Variables	Items	N (%) or Mean ± SD ^1^
Age range (65–95)		77.29 ± 7.7
	65–74	42 (38.9)
	75–85	44 (40.7)
	>85	22 (20.4)
Sex	Male	63 (58.3)
	Female	45 (41.7)
Place of residence	Urban	62 (57.4)
	Rural	46 (42.6)
Cohabitants	Partner or spouse	60 (55.6)
	Other relative	10 (9.3)
	Living alone	18 (16.7)
	Paid carer	5 (4.6)
	Nursing home	15 (13.9)
Clinical history Comorbidities ^2^	<2	36 (32.4)
	x > 2	56 (52.8)
	No comorbidities	16 (14.8)

^1^ SD = Standard deviation. ^2^ Number of chronic conditions potentially disabling according to the classification of the Andalusian Healthcare Service [24].

**Table 2:** Association between predisposing factors of ADOP and ADOP during hospital admission.

Variable	N (%)	No ADOP *n* (%)	ADOP *n* (%)	Chi^2^/Fisher (*p*)
Age				
65–75	50 (46.3)	42 (55.3)	8 (25)	0.001
76–85	38 (35.2)	26 (34.2)	12 (37.5)	
>86	22 (18.5)	8 (10.5)	12 (37.5)	
Male sex	45 (41.7)	33 (43.5)	12 (37.5)	0.569
Comorbidity	57 (52.8)	38 (50)	19 (59.4)	0.021
Medical diagnosis of dementia	15 (13.8)	6 (7.9)	9 (28.2)	0.021
Severe dependent (Barthel < 35)	3 (2.8)	0	3 (9.4)	0.000
Moderate dependent (Barthel 35–65)	27 (25)	13 (17.1)	14 (43.8)	
Slight dependent or independent	78 (72.2)	63 (82.9)	15 (46.9)	
Severe cognitive impairment (Pfeiffer 7–10)	6 (5.6)	2 (2.6)	4 (12.5)	0.000
Moderate cognitive impartment (Pfeiffer 3–7)	16 (14.8)	2 (2.6)	14 (43.8)	
No cognitive impairment	86 (79.6)	72 (94.7)	14 (43.8)	
Auditive sensorial deterioration	19 (17.6)	10 (13.2)	9 (28.1)	0.021
Visual sensorial deterioration	8 (7.4)	4 (5.3)	4 (12.5)	
Auditive and visual sensorial deterioration	3 (2.8)	1 (1.3)	2 (6.3)	
Average nutritional status	47 (43.5)	34 (44.7)	13 (40.6)	0.407
Mediocre nutritional status	28 (25.9)	17 (22.4)	11 (34.4)	
Dehydrated	2 (1.9)	0	2 (6.3)	0.086
Daily use of hypnotics	35 (32.4)	22 (28.9)	13 (40.6)	0.477
Previous ADOP	8 (7.4)	3 (3.9)	5 (15.6)	0.048

**Table 3:** Precipitant factors for ADOP.

Variable	Total (%)	No ADOP (%)	ADOP (%)	Chi^2^/Fisher (*p*)
IV cannula	106 (98.1)	76 (100)	30 (96.8)	0.116
IV therapy	56 (51.9)	35 (47.3)	21 (70)	0.035
Urinary catheter	12 (11.1)	7 (9.2)	5 (16.1)	0.323
Oxygen therapy	92 (85.2)	56 (88.9)	19 (65.5)	0.021
Raised bed rails	68 (63)	37 (48.7)	31 (100)	0.000
Use of diapers	66 (61.1)	37 (48.7)	29 (93.5)	0.000
Wet diapers (with ADOP+)	6 (5.6)	0	6 (21.4)	0.557
Fever	42 (38.9)	33 (44)	9 (28.1)	0.124
Pain or discomfort	17 (15.7)	14 (18.9)	3 (9.7)	0.241
Metabolic disorder	51 (47.2)	33 (43.4)	13 (62.5)	0.135
Withdrawal of hypnotics on admission	9 (8.3)	6 (27.3)	3 (9.4)	1.0

**Table 4:** Association between management of ADOP and functional and cognitive deterioration, age, and gender.

Variable		Verbal *n* (%)	Verbal and Pharmacologic *n* (%)	Pharmacologic and Physical *n* (%)	*p*
Barthel Index	Severe dependent	0	2 (10.5)	0	0.511
	Moderate dependent	4 (50)	8 (42.1)	2 (100)	
	Independent	4 (50)	9 (47.4)	0	
Pfeiffer’s SPMSQ	Normal	3 (37.5)	9 (47.4)	1 (50)	0.976
	Slight–moderate	4 (50)	8 (42.1)	1 (50)	
	Severe	1 (12.5)	2 (10.5)	0	
Age	65–75	3 (37.5)	4 (21.1)	1 (50)	0.266
	76–85	4 (50)	5 (26.3)	1 (50)	
	>86	1 (12.5)	10 (52.6)	0	
Sex	Male	2 (25)	9 (47.4)	1 (50)	0.541
	Female	6 (75)	10 (52.6)	1 (50)	

**Table 5:** Patients’ clinical progression during hospital stay.

**Variable**		**NO ADOP *n* (%)**			**ADOP *n* (%)**		**(*p*)**
Length of admission	<7 days	30 (39.5)			4 (12.5)		0.006
	7–14 days	23 (30.3)			19 (59.4)		
	14–21 days	16 (21.1)			4 (12.5)		
	>21 days	7 (9.2)			5 (15.6)		
Early mobilization	First week	49 (64.5)			5 (15.6)		0.000
	Second week	8 (10.5)			4 (12.5)		
	Third week	0			2 (6.3)		
	No early mobilization	19 (25)			21 (65.6)		
Barthel Index (on discharge)	Severe dependent (<35)	4 (5.5)			12 (37.9)		0.000
	Moderate dependent (40–80)	19 (26)			14 (48.3)		
	Independent (>85)	50 (68.6)			3 (10.3)		
Pfeiffer’s SPMSQ (on discharge)	Severe impairment (8–10)	2 (2.6)			8 (27.6)		0.000
	Moderate impairment (4–7)	4 (5.2)			14 (48.3)		
	No impairment (0–3)	67 (91.8)			7 (24.1)		
		**No ADOP *n*/(%)**		**(*p*)**	**ADOP *n*/(%)**		**(*p*)**
		**Admission**	**Discharge**		**Admission**	**Discharge**	
Barthel Index	Severe dependent (<35)	1/2.5	4/5.5		3/9.4	12/41.4	
	Moderate dependent (40–80)	12/15.8	19/26		14/43.8	14/48.3	
	Independent (>85)	63/82.9	50/68.5	0.112	15/46.9	3/10.3	0.01
Pfeiffer’s SPMSQ	Severe impairment	2/2.6	2/2.7		4/12.5	8/27.6	
	Moderate impairment	2/2.6	14/5.5		14/43.8	14/48.3	
	Not impaired	72/94.7	67/91.8	0.00	14/43.8	7/24.1	0.17

## Data Availability

Not applicable.
